# The microbial food revolution

**DOI:** 10.1038/s41467-023-37891-1

**Published:** 2023-04-19

**Authors:** Alicia E. Graham, Rodrigo Ledesma-Amaro

**Affiliations:** Department of Bioengineering and Imperial College Centre for Synthetic Biology, Imperial College London, London, SW7 2AZ UK

**Keywords:** Applied microbiology, Industrial microbiology

## Abstract

Our current food system relies on unsustainable practices, which often fail to provide healthy diets to a growing population. Therefore, there is an urgent demand for new sustainable nutrition sources and processes. Microorganisms have gained attention as a new food source solution, due to their low carbon footprint, low reliance on land, water and seasonal variations coupled with a favourable nutritional profile. Furthermore, with the emergence and use of new tools, specifically in synthetic biology, the uses of microorganisms have expanded showing great potential to fulfil many of our dietary needs. In this review, we look at the different applications of microorganisms in food, and examine the history, state-of-the-art and potential to disrupt current foods systems. We cover both the use of microbes to produce whole foods out of their biomass and as cell factories to make highly functional and nutritional ingredients. The technical, economical, and societal limitations are also discussed together with the current and future perspectives.

## Abstract (web summary)

In this Review article, the authors discuss the potential of microorganisms as a solution to the challenges faced by our food system. Engineered microorganisms can be used to produce enhanced foods and ingredients in a sustainable manner. The technical, economical, and societal limitations are also discussed together with the current and future perspectives.

## Introduction

The current food systems have been pushed to a crisis, as they struggle to keep up with nutrition and protein demand coupled with population growth^1^. All our food systems—agriculture, animal husbandry and aquaculture—are grappling with the degradation of land, climate change and climate disasters, which are set to rise in the future^2^. Although moving towards plant-based foods is less environmentally harmful, it still relies on climate or season and intensive land, water and chemical use^3^. The time for a microbial revolution in food is ripe as microorganisms have the potential to enhance, improve or even replace the currently available alternatives^4,5^. They have been proven to be an ecological and resilient food source, especially when compared to traditional protein sources such as meat^6,7^. Genetic and system design can advance sustainability further when renewable and waste feedstocks are considered^8,9^. Furthermore, they are highly resilient due to their decentralised nature that does not rely on location limitations, such as temperature or weather^10^. Finally, they also have a high nutritional profile^11^, crucial in the face of rising diet-related health epidemics.

Microorganisms are no stranger in the history of food; however, research has lately revealed the vast array of health benefits and ecological savings that can be derived from using microorganisms in food^12,13^. This has led to an explosion in new applications, improvement in traditional practices using state-of-the-art technology^14–16^ and a better understanding of their roles and benefits^13^. Fermentation can be used both directly on foods to improve nutrition, taste or texture^17,18^, as well as used as a production platform to produce value-added ingredients in the food industry^19–21^. Moreover, using fermentation to produce microbial biomass as a nutritional food source is starting to be adopted in both animal feed and human foods^22–24^. However, there are challenges to overcome in each of these applications, including scalability and economic or ecological sustainability. Novel tools can be applied to these fields to enhance and accelerate the development of microbial-based foods and overcome current limitations. This includes high-resolution and high-throughput characterisation of microorganisms^14,25^, as well as genetic and metabolic engineering tools^4^. By engineering and selecting strains, it is possible to improve flavour^26^ and nutrition^20,27,28^ as well as increase sustainability using waste feed or cheap non-competing carbon sources^8,29^. This can contribute to increasing applications and uptake to propel a microbial revolution in food.

**Fig. 1:**
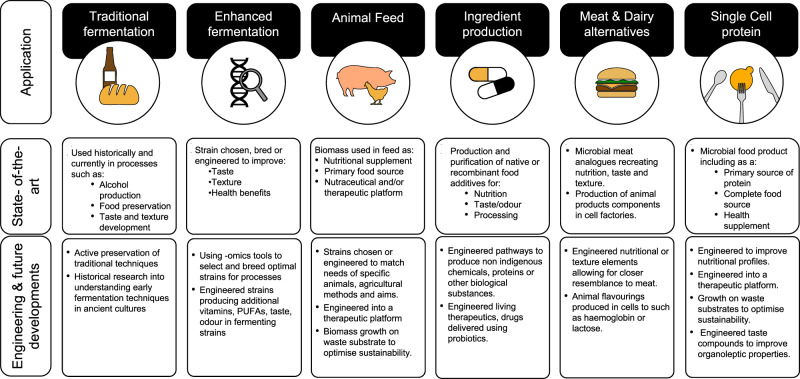
Timeline of the role of microbes in food. A view of the various applications that rely on microbial processes. State-of-the-art in each process is explained as well as the current or potential role of genetic engineering and other future developments to enhance the process or use.

Due to the high potential and varied applications of microbes in food, there have been numerous recent start-ups in this space, ranging from improving traditional fermentation to creating new products (Table 1). Development is still needed for technical advances and consumer acceptance but the field of single-cell proteins and engineered microbes in food has high potential, as will be explored in this review. This review aims to give an overview of the different applications of microorganisms in food ranging from traditional fermentation techniques to biotech applications of ingredient production (see Fig. 1). It covers the different novel applications of microbes in the food system as well as the role of synthetic biology in advancing this field. Finally, the obstacles and future perspectives will be considered.

## The use of microbes in food

### Rise of fermentation in history

Microorganisms were first leveraged by humans in the food system for fermentation. Fermentation is one of the earliest known food technologies dating as far back as 7000BC or earlier and arising independently in multiple ancient cultures^30,31^. Alongside smoking and salting, fermentation was a primary method of food preservation and thus a crucial technology in the rise of human civilisations^32^. In addition, the process also introduced many new products, flavours and tastes. Different fermented products rose from specific environments and conditions which produced a diversity of edible products^32^. These include, but are not limited to, dairy products such as cheese and yoghurt, alcoholic products such as beer and wine, fermented bean products such as soy sauce, douchi (豆豉) and natto, other vegetables such as sauerkraut and kimchi and many more^32^.

The advent of new processing and preservation methods such as refrigeration, the use of natural and artificial preservatives, and freezing and vacuum sealing, among others, have provided alternatives to traditional fermentation. However, more recently, research has brought to our attention the many health benefits offered by a microbial presence in food^13,33^, causing a resurgence in popularity, and many newly popularised health foods are fermented or have fermented ingredients. This is compounded by the rise of plant-based diets and increasing access to international foods—many of which include traditionally fermented products. A good example is Kombucha, a traditional Manchurian fermented tea drink which was introduced to the international market with many purported health benefits and now is valued at over 1 billion US dollars^34^. Other well-known examples are Tempeh and Tofu, two fermented soybean products from Indonesia and China, respectively, which are now consumed as meat-alternative protein sources globally^35^.

### Different functions and health benefits of fermented foods

Fermentation, in the context of food, refers to raw material undergoing enzymatic conversions in the presence of microorganisms^13,36^. These conversions result in alteration in their physicochemical properties. Many of the resulting metabolites play an active role in food preservation, inhibiting the growth of contaminating or spoiling pathogens and increasing shelf life, but others contribute to nutrition, texture, taste and smell^13^. Depending on their composition, fermented food may also bring health benefits. The list is a brief summary of some of the most relevant benefits, although comprehensive reviews can be found on the topic^18,37^:

1. Microbiome enhancing (or probiotic) qualities: The gut microbiome is increasingly proving to be crucial for maintaining health^38^. The use of probiotics supplements has become widely adopted, although the health benefit and strain formulation remain controversial topics^39^. The consumption of certain fermented foods themselves has proven to have probiotic and health-promoting effects^40^.
2. Increasing bioavailability of nutrients in food: This is due to microorganisms breaking food down for easier digestion and absorption of ingested nutrients. For example, lactic acid fermentation can increase the food’s iron content by optimising pH and acid content for solubility^41^. Similarly, fermentation can improve the nutritional value of food by interfering with anti-nutritional factors, which impede protein, carbohydrate or phytochemical availability. For example, trypsin inhibitors found abundantly in various cereals, grains and legumes have been shown reduced activities in fermented foods^42^.
3. Reducing Glycaemic Index: The Glycaemic Index (GI) measures how quickly carbohydrates in food raise blood glucose levels^43^. Probiotic and/or fermented cereals, pseudo-cereals and dairy products have been linked to a reduction in the GI of the food and the blood sugar response^43,44^. Lowering GI intake and response has been shown to reduce risk factors for diseases such as type II diabetes and cardiovascular disease^43^.
4. Removing toxins: Microbial consortia can also act by removing toxic compounds and inhibiting the growth of pathogenic species. For example, Aflatoxin, a common toxin found in foods contaminated with *Aspergillus flavus*, has been shown to be enzymatically reduced in various fermentative processes^45^. Free radicals in vegetable and fruit products are also reduced during fermentation^46^.
5. Biochemical pathways producing health-promoting compounds: Many microorganisms naturally produce nutritionally beneficial chemical compounds including but not limited to antioxidants, polyunsaturated fatty acids, conjugated linoleic acids (CLA), sphingolipids, vitamins and minerals^4,47,48^.

However, fermentation does not always improve the foods and undesired microorganisms can negatively impact some nutritional aspects. Some examples include the production of toxic biogenic amines by lactic acid bacteria^35^, including an increase of free histamine due to the high presence of histidine-producing enzymes (l-histidine decarboxylase) in microorganisms^49^. To counteract this, strategies have been developed to either optimise strain selection^50^ or use engineered strains to enhance biogenic amine degradation^51^. Finally, it is also worth noting that many health claims related to fermented foods are yet to be fully verified by randomised controlled trial studies and have often been exaggerated for marketing purposes^52^.

### The nutritional profile of microbes

**Fig. 2:**
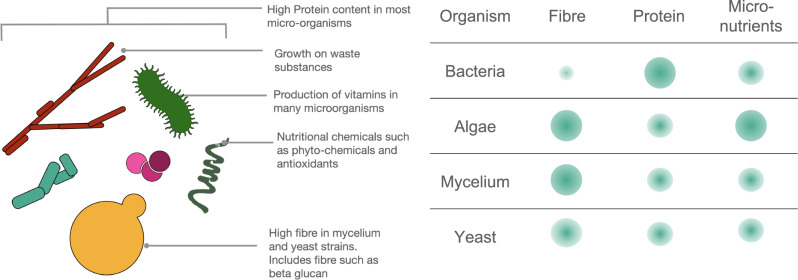
Nutritional profile of microbes. The left panel shows the various components of microorganisms that are beneficiary for nutritional needs. This includes both macro-molecular elements such as proteins and fibre as well as small bioactive compounds. The right panel shows the relative levels of fibre, protein and micronutrients in four groups of microorganisms commonly used for food applications based on comparisons from the review by Ravindra^11^.

Microbial biomass itself also often has qualities that lend itself to consumption as food, including high protein, fibre and bioactive compound content (see Fig. 2).

All microorganisms are generally characterised by high protein content, with algal species averaging between 40–60%, fungi 30–70% and bacteria averaging between 53 to as high as 80%^11,12^. Furthermore, many species are complete amino acid sources, containing adequate amounts of essential amino acids which humans cannot synthesise and need to acquire from diet^53^. In addition, many microbes have a high content of essential amino acids that are lacking in plants^54^.

Fibres, resistant carbohydrates that are key in maintaining gut health^55^, are also elevated in many microbial species^11^. Algae, for instance, has a high fibre content that is composed mainly of insoluble fibres, cellulose and other polysaccharides found in their cell walls^56^. Both filamentous fungi and yeast have potentially beneficial fibres, namely ${{{{{\rm{\beta }}}}}}$-glucan and mannan-oligosaccharides, both of which are consumed as health supplements for gut health and immune-boosting effects^57,58^.

Although lipid content is generally low compared to animal products, oleaginous yeasts and algae are a source of high-value dietary lipids, especially long-chain polyunsaturated fatty acids^34,59^. Interestingly, the overall calorie content can be quite low, such as in commercially available nutritional yeast flakes, which contain 400 calories per 100 g, bringing a high ratio of nutrition to energy. Finally, microorganisms often have high endogenous contents of nutritionally relevant compounds, including vitamins, minerals, antioxidants and other functional ingredients^11^.

The nutritional profile of microorganisms requires further investigation as their use becomes more widespread. The true digestibility of the elements discussed above has not been fully elucidated^11^ and the compositions can differ widely based on different species and the environments in which they are grown^60^. Species need to be carefully selected as some microorganisms also have significant safety and health detriments. An elevated RNA content is often seen in microorganisms which can lead to health issues, such as gout and kidney stones^61^. Some fungal and bacterial species also produce allergens and toxins and are thus ill-suited as food or require processing before ingestion^11^. By carefully choosing species, substrates, and conditions, the nutritional aspects of the food can be modulated to suit specific needs.

## New technologies and applications for microbes in human food

### Enhancing fermentation

Fermentation can be optimised by specially selecting, breeding or engineering strains of microbes to enhance the appearance, taste or health profile of fermented foods^18,62,63^. Traditionally, breeding and selection techniques were used to select for favourable qualities even before the biology of microbes was discovered, leading to vastly different strains for specific uses^30^. Using genetic profiling techniques and -omics technology, we are now able to further identify strains with favourable properties^14,15^. Large-scale analysis has also enabled the identification of strains with desired aromas, which were further improved by hybridisation techniques^16^.

More recently, fermentation has been enhanced by using genetic engineering, where strains used in traditional fermentation can be manipulated to produce additional beneficial products. Some examples of modifications include the enhanced production of B vitamins in *Lactobacilli* used in dairy products^63,64^ or the synthesis of aroma compounds in *S. cerevisiae* strains for novel and improved beer flavours^65^.

Genetic engineering has also been used to improve the sustainability of the fermentative food processes, which can be achieved by expanding or improving substrate range and utilisation^22,66,67^ This furthers the potential to use waste feedstocks^8,9^ and move towards a fully circular economy.

It is worth noting that many fermentation processes are carried out by microbial communities rather than single strains, which adds an additional layer of complexity to the understanding and limits our capacity to improve them. Advances in sequencing technologies and systems biology have allowed us to improve our knowledge of microbial consortia, including those found naturally in foods, as has been reviewed in previous works^68,69^. In addition, in the last years, synthetic biology tools specifically developed to engineer microbial communities have been created^70^, which have the potential to be used to improve food manufacturing. This includes spreading metabolic burden such as when two strategies to reduce browning in soy sauce production were engineered to act synergistically in two microbial species^71^, or enhancing natural coculture properties, such as increasing quorum sensing mechanisms which reduce food spoilage^72^.

### Use of microbes as a protein source in human food

The use of microbes as a food ingredient is known as single-cell protein (SCP) and usually refers either to dried or processed microbe biomass or to the proteins extracted from it. It can be ingested either as a supplement, ingredient or as a main food source (see Fig. 1). Thanks to its potential for sustainable fermentation^8,28^ and its favourable nutritional profile^11^, it has the potential to become a large component of our diet.

SCP has a long and varied history, beginning before the World Wars and continuing into the late and mid 20th century^73,74^. However, most projects were discontinued in the face of rising energy costs and the success of the green revolution, although some legacies remain^75^. One of the first of these is Marmite, established in 1902 as a by-product of the beer industry has even been consumed as an army ration as a source of B vitamins^61^. Since then, there has been development in other, more texturised SCPs—notably that of Quorn. Quorn, established in the 1980s, produces SCP from the filamentous fungi *Fusarium venenatum* and then treated to remove excess nucleic acid content and finally texturized to create meat replacements^76^. It is now a widely distributed product sold in 17 countries with a reported revenue of 236 million GBP in 2020. SCPs are also consumed as a health supplement, such as the microalgae Chlorella and Spirulina, which are rich sources of proteins as well as phytonutrients and vitamins^77^.

Given the ecological and nutritional benefits of SCPs, there is a renewed demand which has resulted in research into new sources of SCPs as well as novel cultivation methods. There is a profusion of start-up companies trying to bring new SCP products to market with some examples listed in Table 1, with many start-ups focussing on meat alternatives.

**Table 1:**
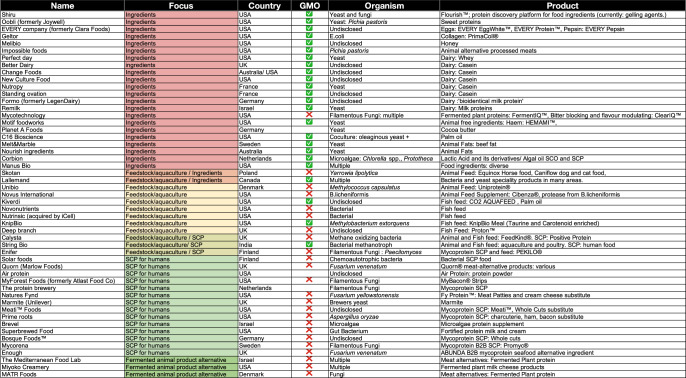
Start-ups and companies developing microbial food either for humans or animal feed as well as individual components or flavourings used

The GMO column has been left blank where unknown.

Different focuses are highlighted with different colour.

So far, most research has focused on wild-type (non-engineered) strains, which have been selected based on their protein content and whose production have been optimised manipulating growing conditions. Synthetic biology has the potential to engineer selected strains to further improve protein production, which can be achieved by (1) enhancing and expanding the capacity to efficiently use desirable feedstocks, (2) improving yields for biomass and protein production and (3) adding functionalities to the single-cell protein by the co-production of valuable compounds such as vitamins or antioxidants^78^. Improving growth and substrate use can greatly improve ecological and economical aspects, for example, by transforming waste into proteins^79^.

### Animal meat alternatives

Microbes are a promising substitute for meat products. This is thanks to their matching protein and nutrient levels, as well as their potential to be modified and texturized to resemble meat.

One of the most established companies is Quorn, which produces SCP derived from filamentous fungi. Quorn has products that resemble meat products from chicken nuggets to beef mince and has a large selection of different textures and forms it comes in refs. ^80, 81^. To achieve this, the long strands of hyphae are mixed with binding agents and then this fibre–gel complex is freeze texturised which allows for hyphal laminations that recreate the fibrous texture of meat^80^. Other start-ups including Meati Foods, Mycorena and Nature’s Fynd are also producing meat analogues from filamentous fungi.

Besides mimicking the nutritional profile or protein content, meat flavourings can also be produced by microbes. These products can be extracted and purified, or the whole microbial biomass can be used. For example, in the Impossible burger, *Pichia pastoris* is engineered to produce soybean leghaemoglobin c2^26^, which recreates part of the flavour profile of meat. The engineered microorganism is then incorporated with other ingredients including soy and potato proteins. Haemoglobin is also being produced as a stand-alone ingredient to add to plant-based meats, such as in the start-up Motif Foodworks. In academia, there is a concentrated effort to produce many variations of haemoglobin proteins which could account for future taste expansions^82^. Other individual components of meat can also be produced, such as the structural elements gelatine and collagen^83,84^.

Finally, one main challenge of recreating meat is providing an adequate lipid composition and content. Most plant-based alternatives utilise plant oils, which have a strongly differing taste and mouthfeel. The endogenous contents of lipids in microbes also differ significantly from that of meat; however, there is vast academic research on producing dietary lipids in microbes. Oleaginous species have been found to be a suitable production platform for highly nutritious fatty acids, such as omega-3 fatty acids which are found abundantly in fish^27^. Furthermore, advancement in the production of microbial oils gives us the potential to not only tune lipid composition but to also modify fatty acids to become more suitable for animal replacement uses^85^. Little focus has been given to mimick animal fats in academic research, although start-ups such as Melt & Marble and Nourish Ingredients aim to make dietary fats for animal replacements through fermentation.

### Other animal product alternatives

Engineering microbes also have the potential to recreate animal products such as dairy and eggs. This is done through precision fermentation, where the pathways of individual components have been engineered into microorganisms.

Milk is composed of oligosaccharides, fats, sugars and proteins, primarily that of casein and whey^4^. These various components are being reproduced using synthetic biology in microorganisms^4^. The main milk proteins, namely casein proteins and whey proteins, have been successfully engineered into various organisms, including bacteria and yeasts^4^. These technologies are being employed by various start-ups developing animal-free milk, such as Perfect day, Better Dairy and Formo, which use purified milk proteins extracted from microbial cell factories and mixed with other fats and sugars.

Human breast milk has also been researched as it is thought to have important effects on the development of the neonatal gut flora and immune system^86^. Components such as milk fats and milk oligosaccharides have been developed with precision fermentation for human breast milk, both in academia as well as in industry, such as by the SME Conagen. Human milk oligosaccharides (HMOs) have been produced in both *S. cerevisiae* and *B. subtilis*^87^ and human milk fats in the oleaginous yeast *Y. lipolytica*^88^. The probiotic effects can also be mimicked by recreating the microbiome of breast milk through the addition of microbial populations to formula^89^. The actual effects of these supplements would benefit from further studies in humans.

Eggs have a larger and more complex group of proteins that are responsible for their unique texture and taste. However, there have been efforts to recombinantly express different proteins, initially for allergenicity and protein studies^90,91^, and more recently as food ingredients^92,93^. Furthermore, there have also commercial efforts to produce egg alternative products made up of multiple egg proteins. This includes the start-up EVERY, which launched an egg white product made from recombinantly produced proteins in 2021.

One animal-based ingredient that has already been largely replaced by precision fermentation is rennet, an enzyme mixture containing chymosin found in the lining of the stomach of young ruminants. Commercial chymosin is now mostly produced in *Aspergillus niger*, which has allowed many kinds of cheese to become suitable for vegetarians as well as reducing the price, benefiting cheese makers^94^.

### Microorganisms in animal feed

The use of microbes in animal feed first appeared over a century ago when brewery by-products were used to supplement feed by Max Delbruck. More recently, using microorganisms as a main or supplemental nutritional source has become established as an industry norm in both animal agriculture^95^ and aquaculture^23^. This is due to an increase in regulatory ease and technological capabilities as well as growing pressures for cost and ecological efficiency^96^.

Many different microbial species have been investigated for the benefits in both animal health and production output^23,24^. Different microbial species each have their own limitations and advantages and thus need to be matched to desired functions and livestock^23,24^. Furthermore, there are different delivery options- including as a sole nutritional source^23^, as nutritional additive^24^ or can act as probiotics^97,98^.

Live microbial supplements can act as probiotics and can either be species delivered to colonise the gut and integrate to improve the existing microflora, or to help balance the existing microbiota by modulating the pH, feed existing microorganisms and to defend against pathogenic species. Using probiotics in animal feed is becoming an industry norm as it has large therapeutic gains while reducing the need for drugs and antibiotics. In addition, the use of probiotics is shown to improve feed uptake, immune response and stress tolerance^97–99^. It has also been linked to increased growth, biomass and milk production^97^.

The new generation of SCP-based animal feed uses engineered microorganisms nutritionally tailored to the target animal^28,78,100^. Moreover, it can be also employed as a nutraceutical and therapeutic platform such as in the previously commercial omega-3 enriched Yarrowia biomass employed in Verlasso® salmon^101^, and the efforts in the start-ups such as Cyanofeed (see Table 1). Vitamins, fatty acids and phytonutrients have been successfully delivered through feed^28^. Finally, engineering organisms to utilise waste substances as carbon sources can greatly lower the ecological footprint of highly polluting animal agriculture industries^28,29^.

## Precision fermentation of food ingredients and additives

One of the most developed uses of engineered microbes in our current food ecosystem is the production of ingredients and additives. For decades, microorganisms have been selected and improved to maximise the synthesis of molecules of interest, first by random mutagenesis and selection and then by genetic and metabolic engineering in a practice called precision fermentation^16,21^. A paradigmatic example is the production of vitamin B2, where chemical synthesis was substituted by fermentation in the 90s^102^. The yields and productivities of the processes are key to determining economic feasibility and therefore, metabolic engineering is playing an important role not only in increasing yields but also enabling the production of heterologous chemicals^22^. Interestingly, the use of genetically engineered strains to produce specific compounds is generally well accepted by consumers. This is because, by the end of the fermentation process, the molecules of interest are extracted and purified. They are therefore typically free of recombinant cells or DNA, allowing them to be labelled as natural products^103^.

While most nutraceuticals and additives with health benefits are still made by chemical synthesis or plant extraction, an increasing number of them are now bio-manufactured by microorganisms^4^. Some of these nutraceuticals include water-soluble vitamins (vitamin B complex and vitamin C) as well as fat-soluble vitamins (vitamin A/D/E and vitamin K)^20^. Other nutraceuticals made by engineered microbes have been reviewed elsewhere^21^, and the list includes omega-3 fatty acids, polyphenols such as resveratrol and naringenin, carotenoids such as beta-carotene or Astaxanthin, and non-proteinogenic amino acids such as GABA and beta-alanine. Other ingredients made by microbes are intended to improve the organoleptic properties of the food to which they are added to, improving taste, odour, colour and feel. Flavour enhancers such as glutamate (MSG), inosine monophosphate (IMP) and guanosine monophosphate (GMP) are made by microbes and contribute to the desired umami flavour^104^. Microbes have also been engineered to produce sweeteners such as stevia-derived molecules, xylitol or erythritol^105–107^. More exotic, hoppy flavours have been engineered into yeast to make tastier beer^65^. Odours and aroma compounds have been made by microbial processes like those of rose (2PE)^108^, orange/lemon (limonene)^109^, mint (menthol)^110^, peach (gamma-decalactone)^111^, among many others.

In addition, coloured molecules have been synthesised by microbes with the intention to be used as pigments for food and beverages. Some examples include orange (beta-carotene, canthaxanthin), red (lycopene, astaxanthin, prodigiosin), yellow (riboflavin), blue (phycocyanin), purple (violacein) and black (melanin) colourants^19^.

## Obstacles and future perspectives

### Technical obstacles

To have a fully incorporated use of microbes in food, there are some technical difficulties that must be overcome. First, one of the main nutritional drawbacks is the high content of nucleic acids—namely RNA content. Ingestion of excessive quantities of nucleic acids particularly purines, increases the quantity of uric acid in the body which is a risk factor for gout and renal calculi as well as a strong risk factor for Metabolic Syndrome and cardiovascular disease^112^. This can be partially mitigated through processing methods, including heating and purification as employed by current single-cell protein manufacturers^113,114^. In the future, it would be possible to envisage an inducible method engineered into microbes to self-purify excess nucleic acids.

As a sole food source, the odours and textures of pure microbial cell mass have been postulated to be unsuited to human palate, however this setback could be improved through breeding or engineering in taste with genetic modifications or by creating mixtures or co-cultures to have novel and pleasant tastes^16,115^.

Many microorganisms, especially yeast, fungal and algal clades also have thick cell walls. In many cases, this is an important contributor of fibre in the diet. However, for some SCP, the thick cell wall can limit the number of nutrients that can be taken up and can itself be indigestible. Therefore, it may be necessary to treat the SCP using heat and/or mechanical and enzymatic processes, improving nutrient bioavailability^114^.

### Food safety

Microbial-based foods and ingredients must go through regulatory approvals, which are stricter when new or engineered species are used. Regulatory bodies assess safety and authorize foods in a country-specific manner. For example, the FDA and EFSA are the main regulatory bodies in the USA and Europe, respectively. Some strategies to facilitate the obtention of approvals for microbial foods include the use of approved organisms and processes, limiting the application to animal feeding, purification of products, and removing foreign DNA and living cells.

The safety of the foods must also be considered for each different species. There has already been extensive investigation into some of the main target species that have confirmed their food safety both for fermentation, ingredient production and SCP use. Special attention must be paid to possible contamination in the process and to the potential production of endo and exotoxins that cause allergic and adverse reactions when ingested. Some toxins may be removed by simple heat or chemical treatments. However, through stringent strain selection^116^, strain engineering^117^ and correct fermentation technologies, contamination and toxin production can be prevented or eliminated.

### Consumer acceptance

One of the largest challenges of deploying single-cell proteins and genetically engineered microorganisms in food is consumer acceptance. Genetic modification is still under strict regulations, which differ between countries with some being particularly strict on introducing food with modified genetic information. Moreover, a large percentage of people still do not accept the idea of eating genetically modified materials. With the increasing awareness of improving the ecological aspect of diets^118^, this attitude might be changing as seen with the popularity with lab-grown meats and some synthetic meat and milk alternatives; however, these products are still uncommon in a commercial setting and therefore not incorporated in the average household’s diet.

To promote consumption, it is thus crucial to take the preparation and cultural context of microbial foods into account. Education and marketing can help counteract unfamiliarity and lack of consumption experience^119^. In addition, the design of microbial foods should consider the need to fulfil religious or cultural values, such as kosher or halal requirements^120^.

### Economic barriers

A large problem of deploying SCP is the capital expenditure needed to expand the technologies and market the new food source. Maintenance costs and substrate usage also limit profitability. Because of the costs incurred for prototype development, one of the initial SCP projects by Imperial Chemical Industries (ICI) was abandoned when it failed to compete with cheap agriculture, especially with modified soybeans^10^. However, more recent technologies seem to suggest that building a plant for growing microbes could now be economically feasible^121^, which is facilitated by the optimisation of the growing conditions^122^, advanced fermentation technologies^123^, and higher yields achieved by engineered microbes^100^. Another economic barrier for commercialisation is the lengthy and expensive process associated with obtaining the necessary regulatory and safety approvals. Although dependent on price, variety and transportation, the employment of waste streams also has the potential to lower the process cost and simultaneously increase sustainability^10^. However, this is harder to introduce to the market as it is not fully understood whether the nutritional qualities of the product would be affected.

## Conclusion

Taken together all the information discussed above, there is an obvious interest in developing more microbial-based foods and ingredients, as seen by the increased number of related academic publications, conferences, companies and commercial products. This is in part encouraged by the consumer demand for healthier and more sustainable foods.

**Fig. 3:**
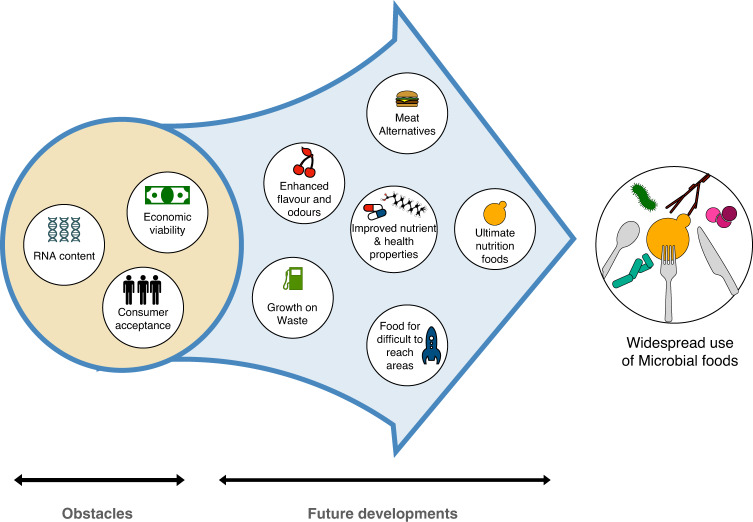
The future of microorganisms in food. A schematic showing the obstacles and future developments in the path to adopting widespread use of Microbial foods. In the beige circle the main obstacles are shown, including the economic viability of some processes, the consumer acceptance of some products, especially GMOs and, in some cases, the presence of undesired molecules. Future developments, shown in the blue arrow, aim to improve microbial-based foods and overcome these obstacles, and include producing nutritionally complete whole foods, alternatives to animal products (meat, dairy, eggs), and ingredients (like flavours or nutraceuticals) that can be made in an affordable and sustainable way, perhaps using waste or CO_2_ as carbon sources.

Synthetic biology and microbial strain engineering broaden the horizons of microbial foods that can be designed, enabling the creation of desired nutritional profiles, aroma compounds, flavours and textures, all of which can build towards personalised nutrition (Fig. 3). To translate this technological capability into sustainable commercial products, the public perception of microbial foods must continue to change and the legislation must facilitate the implementation of these novel processes while maintaining high safety standards. The expansion and normalisation of microbial foods will increase production volumes, decreasing costs and optimising the efficiency of the technology. Reduced costs can then aid the development of microbial processes in less developed areas of the planet, which often need to improve nutrition. Looking at the future, engineered microbes are expected to play a role in delivering food where traditionally inaccessible, such as in disaster relief, deserts or even in space^124,125^.

In conclusion, if there is continued innovation and microbial foods are designed with sustainability and ethics in mind, they have the potential to revolutionise current food systems. This microbial food revolution could be key in designing future-proof strategies to face the health and environmental challenges of the future.
